# Heritability and Inter-Population Differences in Lipid Profiles of *Drosophila melanogaster*

**DOI:** 10.1371/journal.pone.0072726

**Published:** 2013-08-27

**Authors:** Cornelia J. F. Scheitz, Yu Guo, Angela M. Early, Lawrence G. Harshman, Andrew G. Clark

**Affiliations:** 1 Department of Molecular Biology and Genetics, Cornell University, Ithaca, New York, United States of America; 2 Weill Institute for Cell and Molecular Biology, Cornell University, Ithaca, New York, United States of America; 3 Department of Ecology and Evolutionary Biology, Cornell University, Ithaca, New York, United States of America; 4 School of Biological Sciences, University of Nebraska-Lincoln, Lincoln, Nebraska, United States of America; Virginia Commonwealth University, United States of America

## Abstract

Characterizing and understanding the complex spectrum of lipids in higher organisms lags far behind our analysis of genome and transcriptome sequences. Here we generate and evaluate comprehensive lipid profiles (>200 lipids) of 92 inbred lines from five different *Drosophila melanogaster* populations. We find that the majority of lipid species are highly heritable, and even lipids with odd-chain fatty acids, which cannot be generated by the fly itself, also have high heritabilities. Abundance of the endosymbiont *Wolbachia*, a potential provider of odd-chained lipids, was positively correlated with this group of lipids. Additionally, we show that despite years of laboratory rearing on the same medium, the lipid profiles of the five geographic populations are sufficiently distinct for population discrimination. Our data predicts a strikingly different membrane fluidity for flies from the Netherlands, which is supported by their increased ethanol tolerance. We find that 18% of lipids show strong concentration differences between males and females. Through an analysis of the correlation structure of the lipid classes, we find modules of co-regulated lipids and begin to associate these with metabolic constraints. Our data provide a foundation for developing associations between variation in lipid composition with variation in other metabolic attributes, with genome-wide variation, and with metrics of health and overall reproductive fitness.

## Introduction

Because *Drosophila melanogaster* is an established and powerful model organism for a wide range of biological investigations, progress in the analysis of additional biochemical traits is likely to proceed rapidly [1]–[4]. The study of lipid biology of *Drosophila* also has a long history [5] and we are rapidly gaining knowledge about the role of lipids during *Drosophila* development [6], [7], but we have yet to determine a quantitative global understanding of variation in lipid composition, the genetic means by which this variation is regulated, or its influence on cellular functions.

One area of lipid biology that has been well characterized in ectotherms, including *D. melanogaster,* is homeoviscous adaptation [8]–[12]. This refers to the global adaptation of membrane lipid content after temperature changes by alteration of the ratio of phosphatidylcholines to phosphatidylethanolamines (PC/PE) and through modification of the degree of fatty acid (FA) unsaturation. Both are crucial for acclimation by controlling membrane fluidity [9], [13]–[17]. It is known that there is a baseline ability to acclimate to changes in temperature [13], [18], and there is also evidence that selection acts on this ability, particularly with respect to cold acclimation [14]. While the environmental contributions to acclimation and membrane composition have been thoroughly studied, the heritability of these lipid components has not been analyzed to date. Additionally, there is a lack of knowledge about the biological consequences of lipid variation. For instance, phosphatidylserines (PS) are important for recognizing and clearing apoptotic cells [19], but there is no report of the role of fatty acid (FA) chain length or desaturation on the efficacy of PS on apoptosis. The same is true for the response to phosphatidylinositol (PI), which induces the PI-3-kinase cascade, a pathway that plays vital roles in cell growth and survival [20].

Here we present the most comprehensive comparison of lipid composition across populations of *D. melanogaster* to date and for the first time analyze the heritability of each lipid component. The 233 lipids analyzed span all major lipid classes with FAs of various lengths (odd and even carbon chain lengths) and include different degrees of FA saturation. In this dataset, we detect heritable differences in membrane fluidity and corresponding ethanol resistance across populations. We also correlate the lipid profiles with the abundance of the intra-cellular microbe *Wolbachia*. This analysis points toward a trade-off between gaining odd-chained lipids and being at risk for apoptosis. Additionally, we investigate the structure underlying the lipid network in males and females by analyzing the modular pattern of clustering of correlations and by use of discriminant analysis of principal components (DAPC) we find striking differences that point toward the existence of an additional layer of lipid network interactions in females.

## Results

We isolated total lipid from whole adult flies of 92 inbred lines belonging to five geographically distinct populations (Beijing, Ithaca, Netherlands, Tasmania and Zimbabwe). For each line we extracted lipids from three replicates of groups of 10 adults of each sex (separately). From the initial raw data on 342 lipids, 233 survived a series of quality control filters and were retained for further analysis (see Materials and Methods). Our dataset includes phosphatidylethanolamines (PEs, 33 species), phosphatidylcholines (PCs, 34 species), phosphatidylserines (PSs, 12 species), phosphatidylinositols (PIs, 18 species), phosphatidylglycerols (PGs, 22 species), phosphatidic acids (PAs, 2 species), lysoPCs (9 species) and lysoPEs (8 species), as well as triacylglycerols (TAGs, 61 species) and diacylglycerols (DAGs, 33 species) (see Tables S1 and S2). The excluded lipids belonged to all classes (Figure S1A). Odd and even carbon-chain fatty acids (FAs) were excluded at about equal frequencies (Figure S1B), however considering FA carbon-chain length we find that >50% of lipids with FAs of length C_20–22_ did not pass quality control. This percentage is much lower for all other bins (Figure S1C). A recent report of lipid profiles in *D. melanogaster* by Hammad et al. [21] showed absence of FAs greater than C_18._ However, while C_16_ and C_18_ are the most common FAs in our dataset, we do detect lipid species containing C_20_ and C_22_ FAs (Figure S1C) at similar levels to what has been detected in other studies [16], [22].

### Lipid Data are Consistent with the Established Metabolic Network and are Highly Heritable

**Figure 1:**
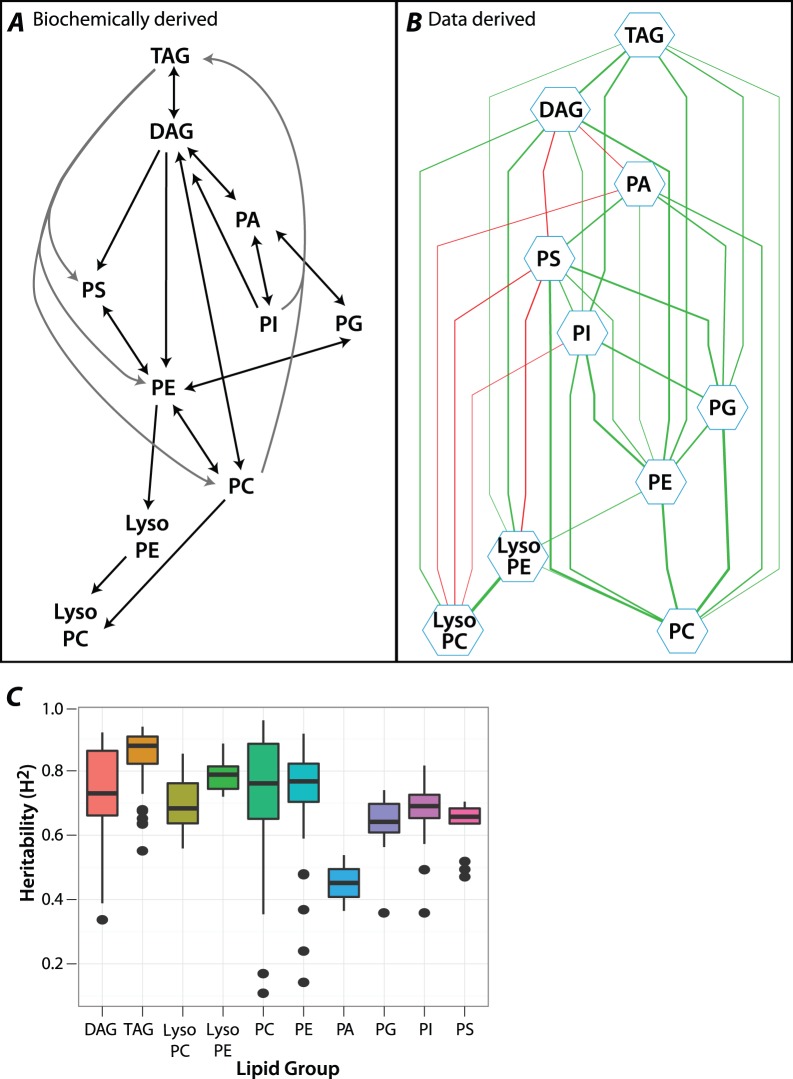
Lipid network conforms with known metabolism and has high heritability. A: Metabolic network for the relations among lipid classes, as derived from the literature [23]. B: Network constructed from our lipid composition data using Cytoscape. All pairs of lipid classes with PCC >0.1 or PCC<−0.1 are connected in the network. A positive correlation between two lipid classes is represented by a green edge, while a negative correlation is represented by a red edge. The thickness of the edges is proportional to the magnitude of PCC between the lipid classes. C: Broad sense heritability (H^2^) for each measured lipid species grouped by lipid class. Circles are outliers and represent specific lipid species.

First we wanted to confirm the quality of our dataset by comparing a network inferred from a simple correlation analysis of our lipid class abundances to known biochemical pathways ([23] and Figure 1A). After removing population and sex effects with linear mixed models (REML) we calculated pair-wise Pearson Correlation Coefficients (PCC) between all lipid classes and constructed a network view of the relationships among these lipid classes using Cytoscape ([24] and Figure 1B). All correlations greater than |0.1| automatically arranged by their cluster distance are displayed, and this network shows broad consistency with the published literature. However, our data suggest additional novel links that remain to be validated biochemically.

Because our measurements were done in a collection of highly inbred lines, we were able to estimate the heritability of the lipids in this network. We calculated the broad-sense heritability (H^2^) for each lipid (plotted by lipid class Figure 1C) by considering variance in line means over the total phenotypic variance (using linear models estimated by REML). There is a high level of heritability across lipid classes, and, with the exception of PAs, the different classes display a considerable range of genetic contribution (Figure 1C). This is the first evidence that variability in lipid composition in *D. melanogaster* is to a large extent mediated by genetic differences among lines. The lipid classes with the most variation in H^2^ are PE and PC and below we detail potential reasons for this pattern.

### Phospholipid Ratios Predict Membrane Fluidity

**Figure 2:**
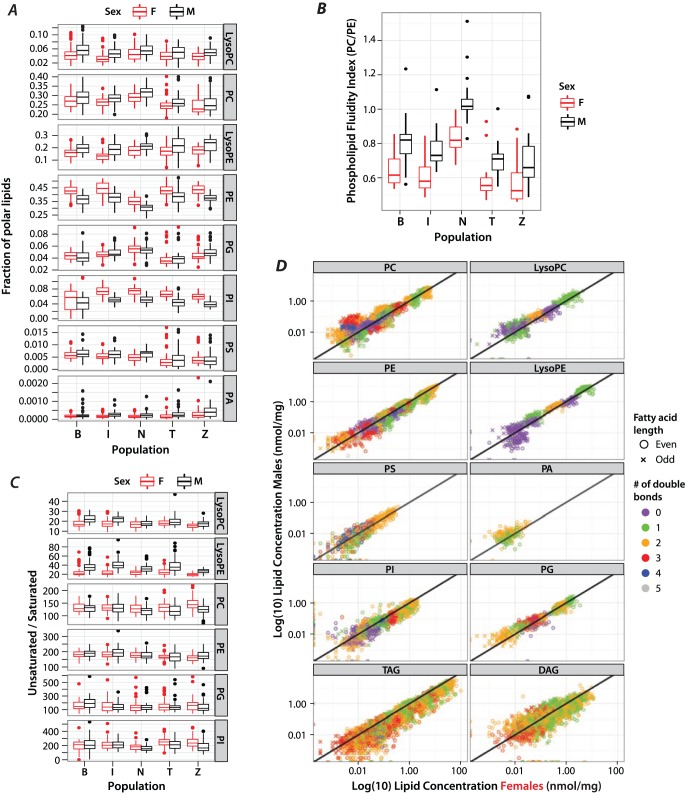
Distribution of lipids and membrane fluidity. A: Proportion of total polar lipids comprised by each polar lipid class in the five populations, stratified by sex. B: Membrane fluidity index as determined by the PC/PE ratio for all populations and sexes. C: Ratio of unsaturated over saturated lipids grouped by lipid class, population and sex. A lipid class was only included if it contained saturated lipids. D: Log-transformed concentration of all lipid species in all lipid classes in males *vs*. females. Samples are color-coded by the number of double bonds and the FA length is partitioned into odd (x) and even (circle) groups.

PE is the primary component of *D. melanogaster* polar lipids at all developmental stages and in all cellular membranes, with the second major membrane polar lipid class being PC [21], [25], [26]. In agreement with this we find that PCs represent ∼40% and PEs ∼28% of all polar lipids (Figure 2A). PEs are generally cone-shaped due to their smaller head-group and they cannot by themselves form a lipid bilayer. On the other hand, PCs spontaneously organize into lipid bilayers due to their cylindrical shape. Together PEs and PCs are the two main structural components of biological membranes. Membranes that contain a low proportion of PE are said to be rigid and are at high risk to transition to the gel phase in cold environments. Adjusting the amount of PE and PC in the inner and outer membrane leaflets is a dynamic and facile way for organisms to adjust their membrane fluidity and prevent this transition. An increase of PE will slightly destabilize the membrane and thus maintain it in the fluid phase. As a consequence, cold-acclimated ectotherms show a lower PC/PE ratio than their warm-acclimated relatives [10]. Additionally, a shift in environmental temperature causes a drop in the PC/PE ratio in several species [27], [28]. Here, we detect a high PC/PE ratio in the Netherlands population, indicating rigid membranes as opposed to fluid membranes in all other populations (Figure 2B). Males also show a slightly higher ratio (more rigid membranes) compared to their female counterparts in all populations (Figure 2B).

A decrease of lipid saturation, summarized by the ratio of unsaturated (US) to saturated (S) lipids, is an additional measure of decreased membrane fluidity [9], [17]. When calculating this US/S ratio for all lipids we excluded all neutral lipids as well as PAs and PSs, because we did not measure completely saturated lipids in these classes. Lyso lipids have the highest degree of saturation across all populations with Zimbabwe flies showing the least unsaturation observed (Figure 2C) due to a loss of species containing a single double bond (not shown). However, striking population differences are not observed across the two major membrane components PE and PC (Figure 2C), although the variation is statistically significant (Table1). While lysoPEs also comprise a substantial fraction of membrane polar lipids (∼19%), it is not clear how much variation in saturation of lysoPEs impacts overall membrane fluidity.

While the US/S ratio is a good simplification of the data, it does not take into account the degree of unsaturation, which clearly also has an effect on membrane fluidity [9], [17]. Thus we contrasted the concentrations of each lipid class in males and females grouped by the number of double bonds, as well as the length of the FA chains (Figure 2D). Generally, males tend to have more highly unsaturated lipids than females for all polar lipids except for PEs and PIs, a finding that is somewhat at odds with a prior study [22].Notice, however, that our results primarily pertain to an increased US/S ratio only in lysoPCs and lysoPEs, which also show the strongest difference across the sexes (Table1). With respect to membrane fluidity, this has the opposite of the effect of increased PC/PE ratio in males (Figure 2B), and thus could serve as a compensation mechanism to maintain membrane fluidity at levels similar to females.

There is little known about the relevance of the specific double bond configuration for each polar lipid class. Our data suggest that highly desaturated lipids are more strongly heritable compared to saturated lipids (Figure S2A), but more research needs to be done to elucidate the significance of this observation. However, we do know that saturated lipids make membranes more rigid and reciprocally unsaturated lipids allow for membrane fluidity. Males have more unsaturated fatty acids overall than females, but females have a lower PC/PE ratio. These two patterns have opposite effects on membrane fluidity, potentially keeping each other in balance. There is no report in the literature of any large difference in membrane fluidity of adult males *vs*. females.

Overall, our findings predict that Netherlands flies have more rigid membranes than any other population tested. One physiological consequence that we might predict from this observation is that Netherlands flies ought to have elevated ethanol resistance. We decided to quantify this directly.

### Netherlands Flies Show Increased Ethanol Resistance

**Figure 3:**
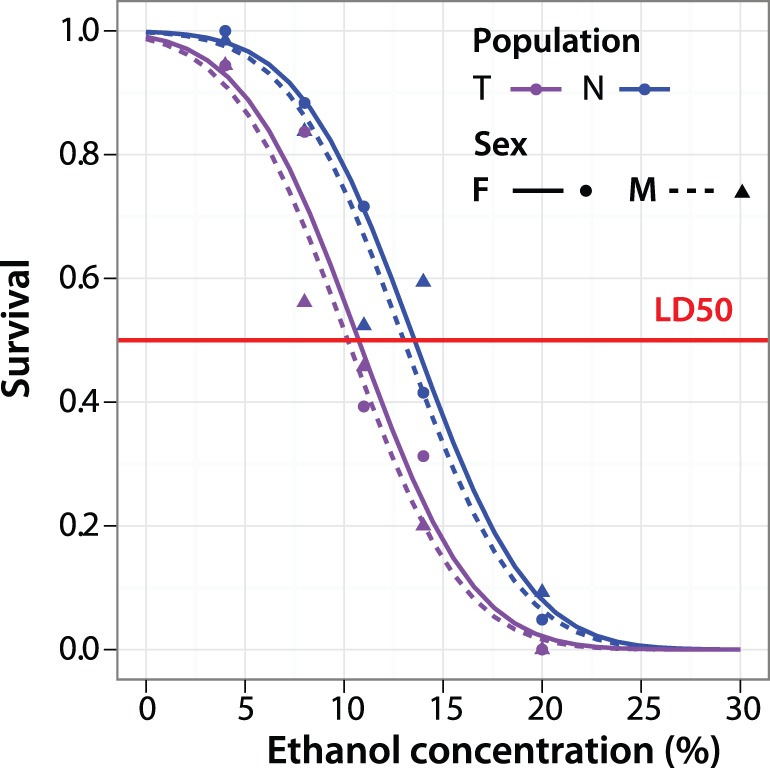
Netherlands flies have higher ethanol tolerance. Ethanol tolerance measured in duplicates for each sex in four lines from both the Netherlands and Tasmania. Red line indicates a survival rate of 0.5.

Previously, Montooth et al. [29] reported that ethanol resistance is correlated to environmental conditions such as rearing temperature, which may mediate ethanol resistance by altering membrane fluidity in flies. To test this hypothesis we compare ethanol resistance in Netherlands to Tasmanian flies. Four lines for each population were randomly selected and reared as before, but instead of extracting lipids we placed flies in vials with sucrose and a graded concentration series of ethanol to determine the lethal dose (LD_50_) after 48 hours. Using probit regression we found that indeed Netherlands flies reach their LD_50_ at around 13% ethanol, while Tasmanian flies show 50% lethality at 10% ethanol (Figure 3). This difference between the two populations is significant with an associated *P*-value of 6.31×10^−11^. This supports the hypothesis that flies with lower membrane fluidity, such as those from the Netherlands, have a higher ethanol resistance. There was no statistically significant difference in ethanol resistance of males and females in this assay. While the PC/PE ratio affects membrane fluidity directly, it may also impact the activity of membrane-associated proteins which in turn may also influence ethanol resistance or cold resistance. For this reason, at this point we cannot distinguish between an indirect and a direct effect of the PC/PE ratio on physiological changes we describe here.

### Minor Phospholipids with a Major Impact on Cellular Metabolism

In addition to the prominent PE and PC lipids PG, PI, and PS lipid classes also show significant variation across populations (Figure 2A and Table 1). These phospholipids are important biological regulators and signaling components. PGs itself have only a few known functions, such as coating lung alveoli in mammals [30] and forming cardiolipins, which are the integral component of mitochondrial membranes anchoring cytochrome C oxidase (complex IV) [31]. Unfortunately, in our ESI-MS/MS, FAs in PGs and TAGs containing 10∶0 form intact ions and neutral fragments of identical nominal masses; hence they are hard to identify with confidence and were dropped from further study.

**Table 1:** ANOVA of lipid species by lipid class.

	Lipid Concentration		Unsaturated/Saturated ratio	
Lipid	Population	Sex	Population	Sex
**PC**	0.4599	8.98×10^−4^	3.01×10^−4^	2.34×10^−7^
**PE**	<2.2×10^−16^	2.35×10^−12^	2.34×10^−7^	7.90×10^−3^
**PC/PE**	<2.2×10^−16^	<2.2×10^−16^	NA	NA
**PS**	2.17×10^−10^	2.21×10^−4^	NA	NA
**PI**	1.87×10^−07^	<2.2×10^−16^	1.02×10^−8^	8.67×10^−4^
**PG**	3.41×10^−08^	0.530	0.058	0.69
**PA**	1.62×10^−13^	3.93×10^−08^	NA	NA
**lysoPC**	2.51×10^−4^	2.61×10^−08^	2.81×10^−16^	5.2×10^−16^
**lysoPE**	3.20×10^−12^	2.66×10^−11^	2.09×10^−13^	<2.2×10^−16^
**DAG-16.1**	<2.2×10^−16^	7.34×10^−10^	NA	NA
**DAG-18.1**	2.20×10^−16^	2.20×10^−16^	NA	NA
**TAG-16.1**	9.80×10^−3^	2.20×10^−16^	NA	NA
**TAG-18.1**	2.40×10^−05^	2.20×10^−16^	NA	NA

A well-known lipid with broad biological impact is PI. Phosphorylation of the inositol ring at the third hydroxyl group by PI-3 kinase is the most common alteration of PIs starting the PI3K/Akt/mTor pathway [32]. This is crucial for many different cellular functions including, cell growth, proliferation, motility and survival, and its mis-regulation is strongly associated with cancer [32]. While a lot is known about the enzymes involved and the downstream players after the initial phosphorylation, it is not known whether the length of the carbon chains in the respective fatty acids influences the efficacy of signaling. In our dataset PI(36∶2) is the most abundant PI and variability across populations within PI is small compared to other lipids, albeit significant. However, it is striking that among the Beijing lines there is a wide variability in PI content (Figure 2A), and inter-line variability is highly significant (*P*-value = 1.37×10^−7^. Among all other populations only Tasmanian flies show a marginally significant among-line variation (*P*-value = 0.041). Overall, the Beijing population would be a good set of flies to study the influence of lipid configuration on PI3K.

Lastly, apoptosis research usually focuses on the enzymes involved as well as factors that mediate apoptotic cell clearance. In mammalian cells that undergo apoptosis, PSs are flipped from the inner membrane leaflet to the outer so that they can be recognized by annexin V thereby recruiting macrophages for cell clearance [19]. While many details are known about the individual proteins, it is not known whether only certain length PSs can be flipped or recognized by annexin V. It is also not clear whether absolute amounts of PIs can serve as an indicator for apoptosis itself or at least the potential for it. All the populations have significant inter-line variation in PIs. More striking is that Tasmanian and Zimbabwean flies have significantly lower PS levels than the other populations (Figure 2A), and this could indicate that these two populations are less primed for apoptosis. Many factors influence the regulation of apoptosis but one is bacteria-induced stress. Both pathogenic and symbiotic bacteria can cause elevation of reactive oxygen species, or increased DNA breaks, resulting in apoptosis [33]. However, symbiotic bacteria also supply unique lipid resources to the host.

### Ratios of Odd and Even Chain Fatty Acids are Environmentally Influenced

Drosophila do not have the capability for *de novo* synthesis of odd-chained fatty acids, but they can acquire odd-chained fatty acids by ingestion of bacteria that do. Drosophila also host a robust gut microflora, and it is likely that this is also a site of production of odd-chained fatty acids. Symbiotic interactions between bacteria and their hosts are highly heritable and this is potentially reflected in the strong heritability of odd-chained lipids (Figure S2B). Interestingly, the statistical significance of heritability of odd-chained fatty acid abundance is higher than that for even-chained lipids (*P*-value = 2.2×10^−16^).

From the ESI-MS/MS results we can extract the total number of carbon atoms within each lipid. In lysophospholipids there is only one FA and so FA length is unambiguously defined. In phospholipids there are two FA chains, hence lipids with an odd total number of FA carbon atoms contain one odd-chain and one even-chain FA, while lipids with an even number of total carbon atoms could contain either two even-chain FAs or two odd-chain FAs. We know that odd-chain lipids are the minority so lipids with an even total are more likely to have two even-chained FAs. The identity of odd-chained lipids was further confirmed with FTICR MS and Q-TOF MS as explained in the Materials and Methods. Briefly, we compared the accurate mass of 12 lipids with the theoretical mass of their odd-chain or esterified even-chained species. For all but one lipid (PE(36∶3) (possibly an PE containing and ether linkage, ePE(36∶3)), the results strongly support the presence of odd-chained lipids (Table S3). We cannot exclude the presence of ether-linked species, but in this data-set odd-chain lipids are certainly present and more prevalent than their ether counterparts. Ether-linked and odd-chain species have been reported separately in *Drosophila* [6], [13], [34], notably, the analysis presented here was carried out in actual samples, suggesting that these different odd-chain lipids can occur simultaneously in one fly. Here, we have calculated the fraction of odd-carbon lipids for each phospholipid class. PA and PS lipids are excluded as no odd-carbon lipids were measured in these two classes.

**Figure 4:**
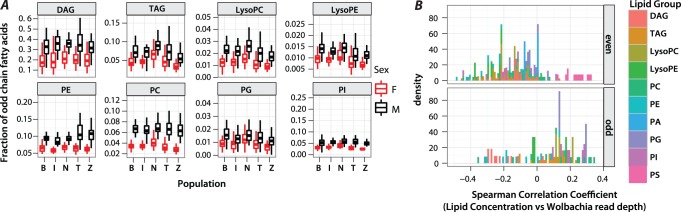
Odd-chain lipid concentration correlates with *Wolbachia* abundance. A: Fraction of odd-chained FAs estimated from the total carbon count per lipid species. Only lipid classes containing odd length FAs are included. B: Histogram of lipid species concentration correlation with normalized *Wolbachia* genome read depth (Spearman) for each fly line where *Wolbachia* was detected (*n* = 65). Lipid species are color-coded by lipid group and split by FA length categorized into odd and even.

While there is no significant variation of odd lipids across populations, it is striking that males have a significantly greater fraction of odd-carbon lipids than females for all lipid classes (Figure 4A). This is due to the increased concentration of mostly even-chained neutral lipids in females, and also to the increased concentration of odd-chained lipids in males across most lipid classes (Figure 2D). Both males and females were reared in the same environment and on the same food, and so differences in metabolism must account for this. Female flies have a larger fat body and harbor more storage lipids to supply the reproductive organs and eggs. However, it is not clear why males would have more odd-chained lipids.

Generally odd-chained lipids originate from external sources such as bacteria, so we next correlated lipid concentration with abundance of the bacterial endosymbiont *Wolbachia pipientis*. We approximated *Wolbachia* abundance by using whole-genome short-read sequencing data, and calculating the ratio of reads that aligned to the *Wolbachia* genome to reads that aligned to the *D. melanogaster* genome. For this analysis we excluded 27 lines that did not contain *Wolbachia*. The genome sequencing data were available for females only. Odd lipids show a positive correlation with *Wolbachia* whereas abundance of even lipids showed no or negative correlations (Figure 4B, *P* *-*value = 7.47×10^−13^), suggesting that *Wolbachia* act as a source of odd lipids, at least in females. In addition, one class of lipids that only contains even-chained FAs is also correlated with *Wolbachia* abundance, PS (Figure 4B), the class responsible for signaling to clear apoptotic cells. Notably, without PS the difference in correlations between odd- and even-chained lipids becomes stronger (*P-*value = 2.20×10^−16^), whereas the distribution of PS does show a slight shift toward stronger correlations (*P*-value = 8.17×10^−3^). This suggests that at least in females while having a new class of lipids available, *Wolbachia* presence also results in a significant amount of stress to the organism, increasing its apoptosis, which is in line with a recent study in *D. simulans* [35]. Furthermore, since all subclasses of PS are positively correlated, our data suggest that they all may play a role in induction of apoptosis.

### Correlation Structure of Lipid Classes and Sexual Dimorphism

Our dataset confirms that females have a large quantity of storage lipids (TAGs see Figure 2D). While all neutral lipid classes show statistically significant population variation (Table 1), the general pattern across the classes is constant. Within neutral lipids males in general have a higher fraction of 16∶1 containing DAGs at the expense of 18∶1 containing TAGs. Across all polar and neutral lipids we find a total of 72 lipid species that show clear sex-differences in their concentration, and 43 of those show females to have the greater lipid quantity. The majority of these lipids belong to the storage TAGs. Notably, some lipids have a reversed pattern in different populations (PIs, Figure S3A). Also high concentrations of, for example DAG(37∶2) (16∶1 containing), are strongly male-biased, whereas other DAG species such as DAG(34∶1) (18∶1 containing) are female biased, as is the entire class of DAGs (Figure S3B). Within the polar lipids PEs and PCs show the most sex-biased abundance. PEs are in general more female-biased, but in PCs this is variable. PC(36∶2) is strongly male-biased, but (PC30∶1) female-biased. Interestingly we observe an additional pattern where the lipid concentration is relatively stable across females, but varies greatly within males (for example PC(32∶2), Figure S2B).

**Figure 5:**
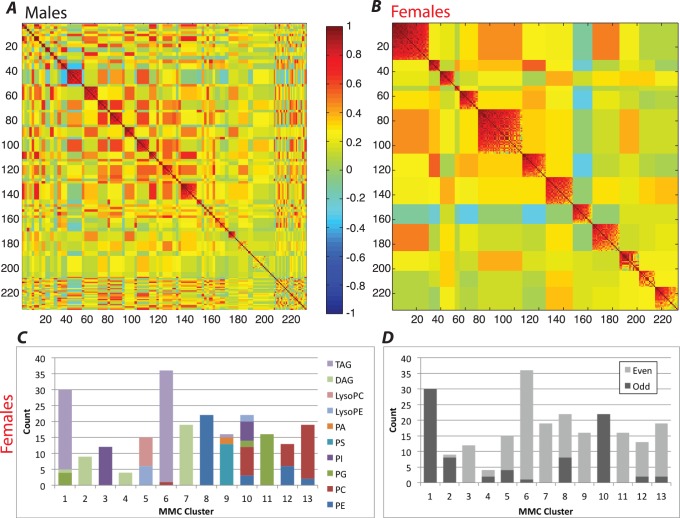
MMC clustering reveals distinct clusters only in female lipid data. A and B: MMC matrix for male (A) and female (B) lipid concentration with any population effect removed. C: Lipid class distribution across identified clusters in females (from B). D: Odd and even chain FA distribution across the same clusters.

In addition to analyzing the individual lipid classes we also determined the clusters of intercorrelated lipid classes that form in each population. To compare sexes we removed the population contribution and grouped lipids using modulated modularity clustering [36], [37]. Strikingly, in the males there were 72 weakly supported clusters, each containing three lipids on average. Hence, there is no significant grouping of lipids observed, not even according to the lipid head groups (Figure 5A). In contrast, in females lipids form 13 clusters mostly determined by lipid class and odd or even chain FAs (Figure 5B). Of particular interest is cluster 10, which contains only odd-chain FAs, but it includes all of the distinct polar lipid classes. This composition is unique among the clusters we find (Figure 5C,D), but the biological significance is not clear. This cluster possibly derives from the fact that odd-chained lipids have to be drawn from a limited pool that is acquired through food or symbiotic bacteria. At this point we cannot determine whether the difference between males and females in odd-chain fatty acids is attributable to sex differences in Wolbachia levels, because Wolbachia levels were not measured in males. Other correlations confirm the top-level structure presented above, such as the negative correlation of DAGs with its derivative lipids (compare Figure 1B to cluster 9 of Figure 5B).

**Figure 6:**
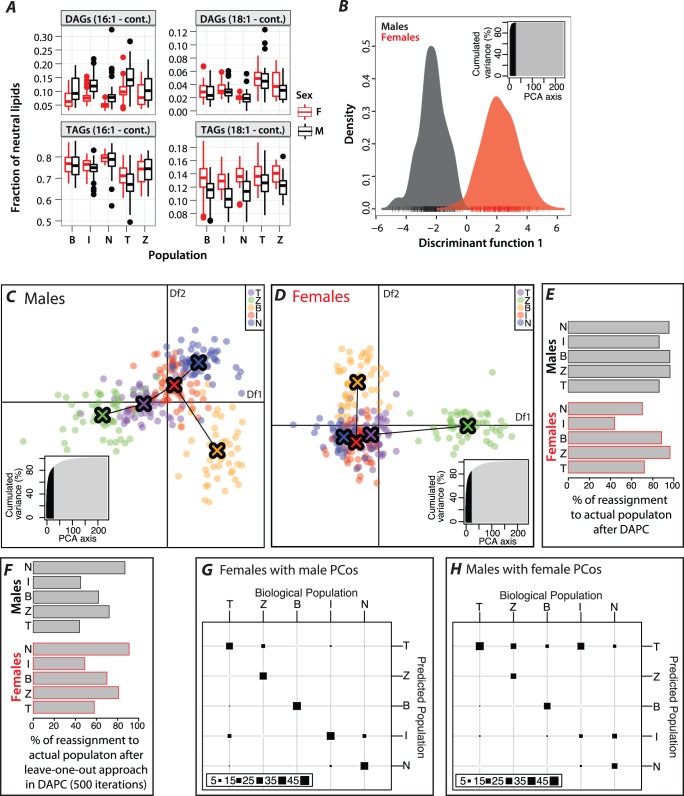
DAPC analysis reveals clear population separation in either males or females. A: Fraction of neutral lipids across population and sex. Value in brackets (cont.) identifies one FA of a total 2 for DAGs and a total 3 for TAGs. B: DAPC analysis for all flies investigating the sex separation. Inset shows the total variance explained by the PCos. Retained PCos are colored black. C and D: DAPC analysis for males (C) and females (D) separating the 5 populations. Colored crosses and their connections show the minimum-spanning tree based on the population distances. Df = Discriminant function. Inset as in B. E: Percent of individuals that are assigned to their biological population using the DAPC of C and D. F: Leave-one-out approach testing the robustness of the population assignment from the DAPC analysis in C and D. G: Eigenvectors from the male DAPC (C) are applied to the female lipid dataset, and it was determined how well this allows for population separation by measuring the re-assignment of individuals to their biological population. H: Same as F, but female derived eigenvectors are applied to the male dataset.

We next carried out discriminant analysis of principal components (DAPC) using our dataset for males and females without the population component (Figure 6B). Retaining 20 principal components (PCos) we see a clear separation between the sexes. The set of lipids with a component loading of greater than 0.2 contains several TAGs and DAGs as expected, but also various signaling and membrane lipids (Figure S4A).

### Discriminant Analysis of Principal Components Reveals Significant Differences in Population Structure between Males and Females

The sex disparities prompted us to investigate population structure in males and females separately using DAPC. To achieve a clear separation of populations we retained 30 PCos in either sex for the discriminate analysis. In both males and females the components explain slightly over 80% of the cumulative variance in the sample (Figure6 C, D). There are prominent differences between the sexes in their population distribution. The males show one clear outlier, the Beijing population, and all other populations are equally spaced from one another (Figure 6C,D). In line with a clear separation, grouping populations according to DAPC matches the original population assignment for at least 80% of lines in each population (Figure 6E). Strikingly, the females show two outlier populations; Zimbabwe and Beijing. However, Ithaca, Tasmanian and Dutch flies map very close together, although still in the same order as in males, resulting in ill-defined populations boundaries between the three of them. The weakest ability to discriminate geographic location was seen with the Ithaca flies, where only 45% of the cases are assigned (Figure 6E). We also tested the robustness of the group assignment in each sex determined by DAPC. We computed eigenvectors and eigenvalues leaving out one line per population and then used the computed PCos where the held-out line would be predicted to fall. The success of the leave-one-out approach is dependent both on the variation within a population (cluster strength) and among populations (cluster distance). In the Netherlands males, for example, the leave-one-out misclassification error rate was less than 10%, meaning that more than 90% of the time, one could successfully identify a fly from this population based only on its lipids (Figure 6F). For each population, females showed a slightly better recovery, indicating that although populations are more overlapping, they are more tightly clustered and thus removing one line will less affect the boundaries defining that group (Figure 6F).

To understand the underlying differences between male and female PCos we looked at the contribution of the individual lipids to the population discrimination. As expected by our previous analysis, there is little overlap between the top contributing lipids between the sexes and neither set of female-or male-loading lipids intersects with the set discriminating sex except for PI(34∶2) (Figure S4A).

While it is expected that lipid biology is drastically different in males and females, we still expected some similarities across populations. Thus we hypothesized that maybe some of the components could also differentiate between the populations in the other sex, but were only secondary to the ones driving the population differences. Consequently we asked if male PCos could recover female population structure and vice versa and the results are summarized in Figure6 G, H and Figure S4B, C. Interestingly, females fall into their biological populations when predicted with male PCos. However, males did not group well when predicted from female PCos, resulting in the misclassification of all populations, especially assigning Ithaca and Zimbabwe lines to the Tasmanian population (Figure 6G). These results suggest the presence of an additional component of lipid biology present in females, whereas the basic lipid biochemistry surfacing in the male DAPC is shared across sexes. This is not unexpected due to the presence of additional fat body and related functions in female reproduction.

## Discussion

We are just beginning to understand the interactions of environment and genotype and its effect on lipid abundance and membrane phospholipid composition. This is the most comprehensive comparison of lipid profiles across natural lines of *D. melanogaster* to date. The lipid species found in our profile are mostly in agreement with a prior report of Hammad et al. [21] using a new LC-MRM approach (liquid chromatography multiple reaction monitoring). Across all lipids C_16_ and C_18_ FAs dominate [13], [21] which in our study translates to dominance of a total of 34 carbon atoms across the FAs within a lipid species. However, they do not find phospholipids longer than C_18_, but we detect small amounts of what have to be C_20_ PC and PE and minor amounts in PI and PS. Additionally we find TAGs with C_20_ and even C_22_ for DAGs. Parisi et al. [22] also detected C_20_ but not C_22_ FAs. All lipid species with very long FAs have small concentrations and other prior studies indicate that these long-chain FAs exist in *D. melanogaster* [16], [22]. Given that Hammad and colleagues used a different detection method, this finding indicates that the LC-MRM approach is biased toward medium and long-chain FAs as opposed to very long-chain FAs and that mass spectrometry could give a more complete profile.

Using this rich dataset we can for the first time assess the heritability of the storage and signaling lipid components in metabolism. Overall, the heritability is very high for all classes, but highest for TAGs, the storage lipid component. Notably, PCs and PEs have both strongly and weakly heritable species. Many factors influence levels of heritability, including varying levels of genetic variation for locally adapted traits, as well as varying levels of environmental impact on the phenotype. At present we cannot yet say which of these is primarily responsible for the variability across species of PCs and PEs, although the role of the PE/PC ratio on membrane fluidity is clear [38].

One of the most striking inter-population differences that we saw was in the PC/PE ratio, where Dutch flies show a clear signature of highly structured membranes as opposed to relatively fluid membranes for all other populations. Phospholipids are necessary for proper signaling but more importantly form the cell membranes, which are the physical barriers between the extra- and intra-cellular compartments [9], [39]. PCs and PEs are the main structural membrane components and they also function as gatekeepers to allow for selective transport across membranes enabling proteins and nutrients to enter the cell [21], [25], [26]. To confirm that Dutch flies have less fluid membranes several methods were available. Phenotypic plasticity in membrane fluidity confers an increased survival rate upon cold-tolerance and cold-shock assays, but the detailed effects of cold-tolerance and cold-shock on membrane lipid composition are vastly different [13], [16], [40]. However, there is no standardized test for cold-shock or cold-tolerance and each proposed test has significant set-up and experimental variation, leading to conflicting results from various studies [16], [41]. In addition, starvation influences the outcomes since cold-treatment and recovery are routinely carried out in empty vials. Thus we used the alternative measure of ethanol resistance to verify our conclusions. Overall, membrane fluidity is influenced by temperature but also dietary constraints. Dietary ethanol will integrate into the membrane and ethanol degradation alters the PC and PE regulation dramatically increasing its fluidity [5], [42]–[47]. Thus individuals with more rigid membranes show a higher ethanol resistance. This assay is easily standardized and the sucrose-ethanol mix provides a basic diet. Our observation that Dutch flies have elevated ethanol resistance is consistent with the hypothesis that they have more rigid membranes, as was predicted from their PC/PE ratio.

These results raise the question of how the PC/PE ratio differences arose in these populations. There are two possibilities a) they can be inherited and remain constant across the transition to the laboratory environment, and b) they may have developed upon the transition of the original environment to the laboratory, and are thus an example of homeoviscous adaptation. Cold-tolerance results in an overall shortening of FAs reflected in an increased ratio of 16∶1 FAs [18], [34] and this effect was also seen in *D. melanogaster* populations along a south-north gradient [18], supporting the argument that these are long-term changes. Unfortunately we cannot distinguish between the individual FAs in a phospholipid to analyze if we see a decreased amount of 16∶1 FAs in Netherlands flies, but the total number of carbon atoms in a phospholipid is not significantly higher in Netherlands flies compared to the other populations. However, the effects of long-term cold-acclimation go well beyond membrane fluidity affecting basic metabolic processes such as cell cycle regulation, the production of anti-freeze proteins and adaptation of protein folding [48]–[52]. In fact these changes can alleviate the requirement for membrane fluidity adjustment. It is possible that our Netherlands population is the only one with the necessary alterations to cope with low temperatures without serious detrimental impact to membrane fluidity. On the other hand Beijing, Ithaca, Zimbabwe and Tasmanian flies adjusted by increasing their membrane fluidity, developing a form of cold-tolerance. In agreement with prior studies of cold-acclimation our data reveals a decrease in PC/PE ratio, but no changes in the US/S ratio [13], [16]. At this point we cannot conclusively determine if cold-acclimation as a result of the shift to the constant laboratory temperature did affect the membrane composition. Alternatively, the difference between the Netherlands and the remaining populations is not a result of cold-acclimation, but may instead be driven my some other physiological adaptation or even be neutral. A study by Cooper et al. [14] showed that long-term rearing of two sub-populations under two different environmental conditions did not lead to a divergence in PC/PE ratio. To identify whether the difference in PC/PE ratio is strictly inherited, due to cold-acclimation or a combination of the two, one would need to determine the lipid composition of wild-caught flies and their transition to laboratory adaptation.

To date there is significantly less known about the biological significance of variation in the other signaling lipid classes PG, PI and PS. Especially PG has been little studied despite its potential for major biological impact. In our dataset we could not unambiguously identify PGs versus TAGs, but considering overall levels, Netherlands flies have the biggest pool of PGs (Figure 2C). Mutants in the cardiolipin biosynthetic pathway suggest that PG and cardiolipin content are positively correlated [31], and thus a bigger pool of PGs could be indicative of more mitochondria, thereby reflecting an up-regulation in energy and heat metabolism. This connection should be evaluated further in the future, as mitochondria are crucial to cells and overall body condition. Similarly, the influence of PI double bond conformation and FA length on PI3K induction is not known, but we find significant population variation. PIs are required to trigger the PI3K pathway, which has been extensively studied in the context of cancer formation [20]. There is strong motivation to fill this gap in our knowledge by adding lipidomics to our research.

Our analysis of *Wolbachia* content is a prime example of how lipidomics can expand our understanding of cell biology and cellular interactions with the environment. Intriguingly, we find that odd lipids are significantly associated with *Wolbachia* abundance, consistent with a role of bacteria in regulating the odd-chain FA supply. This raises the question which other bacteria in *D. melanogaster* provide odd-chain FAs and if there is a dominating species. Whole-fly bacterial concentration measurements would yield a better resolution that can be used to gain leverage at the network that likely exists. Surprisingly our data show that *Wolbachia* load is also positively correlated with PS content, notably a lipid class that only contained even-chained FAs in our dataset. PSs trigger macrophage clearance of apoptotic cells by being presented to the extra-cellular space where annexin V can recognize them. The most straightforward interpretation for the correlation of PS lipids with *Wolbachia* content is that presence of this endosymbiont also causes cellular stress that ultimately results in an increased potential for apoptosis. A similar connection has been made in *D. simulans* where *Wolbachia* presence in spermatocytes was associated with an increase in DNA breaks and apoptosis [34]. Notably, this association of all PS species with *Wolbachia* abundance also suggests that all members of this class can signal apoptosis, disregarding the double bond status or FA length, albeit with different efficiencies. Moreover it proposes that PS concentration could indeed be a measure of apoptosis.

Lastly, we investigated the population structure apparent in the lipid data. A prior report from Parisi et al. [22] reported little difference across the sexes, whereas this study, which also used mass spectrometry, successfully identified many lipids with strong sex differences. The discrepancies in sex differences across lipids are likely attributable to the fact that Parisi and colleagues analyzed a single laboratory strain and our dataset is based on 92 lines with natural variation. In addition to striking high/low concentration differences for individual lipid species across males and females we find that modularity clustering leads to two sharply contrasting correlation patterns. It is possible, that the small clusters found in males are subgroups within the larger clusters found in females. Additionally, DAPC analysis indicates that females have an additional layer of lipid biology that is absent in males. Eigenvectors derived from male lipid profiles can be used to transform the female data, leading to a very similar population separation, however the reverse is not true. This is in line with the clusters observed in the MMC analysis and indicates that the distinct structures in males and females are due to differences across all lipid classes and their interactions. Accordingly, analyzing the strongest differences between the lipid profiles in males and females identifies species of all lipid classes with neutral (storage) lipids showing the greatest differences. This is consistent with females having additional fat body tissue. Overall, this demonstrates that there are more subtle differences between male and female lipid metabolism that have a big effect on the observed population variance.

There remains a great deal to learn about the biological significance of lipid variation. Various forms of “omics” research are coming together to present particularly informative patterns of correlation that link with known biochemistry and propose novel insights. So far research has focused on proteins and their interactions with other proteins, and with DNA and RNA. The analysis of variation in lipids lags behind, but our demonstration of the strong signatures of inter-line variability, of heritability and of modular clustering of correlations beg for a deeper functional analysis.

## Materials and Methods

### Fly Populations and Experimental Design

We obtained lipid measures from 92 lines derived from 5 populations of *D. melanogaster*; Beijing (B), Ithaca (I), Netherlands (N), Tasmania (T), and Zimbabwe (Z). The lines were derived from wild-caught females that were inbred for 13 generations by sib mating (previously described in [53]). Overall, they were grown for more than15 generations at constant room temperature prior to lipid extraction. For the experimental generation all lines were grown on Cornell BLA media at 25°C. Males and Females were separated 3 days after eclosion and reared for another 3 days. Flies were frozen in sets of 10 for lipid extraction, or placed in vials for ethanol resistance measurement.

### Lipid Extraction

Lipids were extracted from 3 biological replicates per fly sex and line using a modified Bligh-Dyer lipid extraction [54]. Briefly, flies were weighed before and after the 5-step extraction. To maintain the signal of polyunsaturated lipids 0.01% butylated hydroxytoluene (Fisher) was added to the chloroform used in each step. The lipid containing chloroform was washed with 1M KCl and water before being transferred to a screw-cap vial and dried down using nitrogen for shipping. The lipid profiles were determined by the Kansas Lipidomics Research Center using ESI-MS/MS.

### ESI-MS/MS Lipid Profiling

Total lipid extraction was carried out according to [55] and lipids were dissolved in chloroform for analysis. The profiles of membrane lipids were measured by an automated electrospray ionization tandem mass spectrometry method as previously described [55]–[58]) or by a slightly modified procedure described here. The samples were dissolved in 1 ml chloroform. An aliquot of 40 µl of extract in chloroform was used. Precise amounts of internal standards, obtained and quantified as previously described [55]), were added in the following quantities: 0.30 nmol di12∶0-PC, 0.30 nmol di24∶1-PC,0.30 nmol 13∶0-lysoPC, 0.30 nmol 19∶0-lysoPC, 0.30 nmol di12∶0-PE, 0.30 nmol di23∶0-PE,0.30 nmol 14∶0-lysoPE, 0.30 nmol 18∶0-lysoPE, 0.30 nmol di8∶0-PG, 0.30 nmol di20∶0(phytanoyl)-PG,0.30 nmol di14∶0-PA, 0.30 nmol di20∶0(phytanoyl)-PA, 0.20 nmol di14∶0-PS, 0.20 nmol di20∶0(phytanoyl)-PS, 0.47 nmol 16∶0–18∶0-PI,0.33 nmol di18∶0-PI, 4.65 nmol di15∶0-DAG, and 3.10 nmol tri17∶1-TAG. The sample and internal standard mixture was combined with solvents, such that the ratio of chloroform/methanol/300 mM ammonium acetate in water was 300/665/35, and the final volume was 1.2 ml.

Unfractionated lipid extracts were introduced by continuous infusion into the ESI source on a triple quadrupole MS/MS (API 4000, Applied Biosystems, Foster City, CA). Samples were introduced using an autosampler (LC Mini PAL, CTC Analytics AG, Zwingen, Switzerland) fitted with the required injection loop for the acquisition time and presented to the ESI needle at 30 μl/min.

Sequential precursor and neutral loss scans of the extracts produce a series of spectra with each spectrum revealing a set of lipid species containing a common head group fragment. Lipid species were detected with the following scans: PC and lysoPC, [M+H]^+^ ions in positive ion mode with Precursor of 184.1 (Pre 184.1); PE and lysoPE, [M+H]^+^ ions in positive ion mode with Neutral Loss of 141.0 (NL 141.0); PG, [M+NH4]^+^ in positive ion mode with NL 189.0 for PG; lysoPG, [M – H]^−^ in negative mode with Pre 152.9; PI, [M+NH4]^+^ in positive ion mode with NL 277.0; PS, [M+NH4]^+^ in positive ion mode with NL 185.0; PA, [M+NH4]^+^ in positive ion mode with NL 115.0; DAG internal standards species containing 15∶0, [M+NH4]^+^ in positive ion mode with NL 259.2;TAG internal standards species containing 17∶1, [M+NH4]^+^ in positive ion mode with NL 285.2; DAG and TAG containing 16∶1, [M+NH4]^+^ in positive ion mode with NL 271.2;and DAG and TAG containing 18∶1, [M+NH4]^+^ in positive ion mode with NL 299.2. The scan speed was 50 or 100 u per sec. The collision gas pressure was set at 2 (arbitrary units). The collision energies, with nitrogen in the collision cell, were +28 V for PE, +40 V for PC, +25 V for PI, PS and PA, +20 V and PG, +20 V for DAG and TAG. Declustering potentials were +100 V for PE, PC, PA, PG, PI, and PS, and +100 V for DAG and TAG. Entrance potentials were +15 V for PE, +14 V for PC, PI, PA, PG, and PS, and +14 V for DAG and TAG. Exit potentials were +11 V for PE, +14 V for PC, PI, PA, PG, PS, and +14 V for DAG and TAG. The mass analyzers were adjusted to a resolution of 0.7 u full width at half height. For each spectrum, 9 to 150 continuum scans were averaged in multiple channel analyzer mode. The source temperature (heated nebulizer) was 100°C, the interface heater was on, +5.5 kV or −4.5 kV were applied to the electrospray capillary, the curtain gas was set at 20 (arbitrary units), and the two ion source gases were set at 45 (arbitrary units).

The background of each spectrum was subtracted, the data were smoothed, and peak areas integrated using a custom script and Applied Biosystems Analyst software. The lipids in each class were quantified in comparison to the two internal standards of that class. The first and typically every 11^th^ set of mass spectra were acquired on the internal standard mixture only. Peaks corresponding to the target lipids in these spectra were identified and molar amounts calculated in comparison to the internal standards on the same lipid class. To correct for chemical or instrumental noise in the samples, the molar amount of each lipid metabolite detected in the “internal standards only” spectra was subtracted from the molar amount of each metabolite calculated in each set of sample spectra. The data from each “internal standards only” set of spectra was used to correct the data from the following 10 samples. We used the previously determined limit of detection (LOD) of 0.002 nmol [59] to filter the lipids for analysis. Finally, the data were corrected for the fraction of the sample analyzed and normalized to the sample “dry weights” to produce data in the units nmol/mg.

For the TAG and DAG analysis mass spectral signals in the NL 271.2 and NL 299.2 scans were normalized to the signal for 4.65 nmol di15∶0-DAG in the NL 259.2 scan (for DAG species), and 3.10 nmol tri17∶1-TAG (for TAG species); relative mass spectral signal/lean weight for DAG was calculated by the following formula:
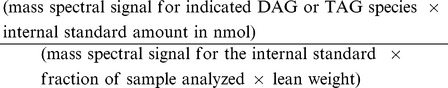



Thus, the data are presented as relative mass spectral signals, where a value of 1 for a DAG or TAG represents the same amount of signal as 1 nmol of DAG or TAG standard, respectively.

### Odd-chain Lipid Confirmation

Calibrated accurate mass scanning (QTOF) and Fourier Transform Ion Cyclotron Resonance mass spectrometry (FTICR MS) were carried out on a subset of samples to support our initial detection of odd-chained lipids. In QTOF positive and negative product ions of single infused samples were scanned for their fragment mass. The experimentally determined values were then compared to the theoretical masses of odd-chain fatty acids and their esterified even-chain counterparts. The positive ion scan was calibrated internally, the negative ion scan externally leading to larger margins of uncertainty which were, however, still sufficient for this experiment. FTICR MS allows for molecular formula determination based on the accurate mass of intact molecular ions. Here a pooled sample was used and a subset of ions (matching the nominal masses in question) was analyzed. The masses of the detected ions were then again compared to their expected masses in either and odd-chain species or the ether-linked species. QTOF MS resolution was 7000, and the resolution of FTICR measurements of intact ions >50,000. Both were easily sufficient for the discrimination among odd-chain-containing and potential alternative lipid molecular species.

### Ethanol Resistance

10–15 flies are placed in vials containing a Whatman disk soaked in 1 ml of a 3% sucrose solution supplemented with 4, 8, 11, 14 or 20% ethanol sealed with parafilm. Duplicate vials were placed at 25°C for 48 hours. Live and dead flies were scored and recorded for statistical analysis.

### Wolbachia Infection Status

In brief, genomic DNA was isolated from 50 adult female flies with Qiagen Blood & Tissue DNA isolation kits. Total DNA was sequenced on an Illumina HiSeq 2000 using paired-end, 100 base pair reads with an insert size of approximately 500. We performed alignments to the *Wolbachia* genome (ASM802v1) with Mosaik v2.1.33 (http://bioinformatics.bc.edu/marthlab/Mosaik/; hash size = 11, max mismatches = 4, alignment candidate threshold = 20, banded Smith-Waterman algorithm). Alignments to the *Drosophila melanogaster* reference genome (release 5.34) were performed using default paired-end settings for BWA [60]. To determine *Wolbachia* infection status, we aligned whole-genome short-read sequences from each of the 92 fly lines to both the *Wolbachia pipientis* (strain *w*Mel) and *D. melanogaster* reference genomes. We then calculated relative bacterial density as the ratio of *Wolbachia*-aligned reads to *D. melanogaster*-aligned reads.

### Statistics

The raw data were normalized and lipids lacking data or significantly deviating from a normal distribution were excluded (*n* = 109). Additionally, one replicate had an ∼8 fold increased concentration for each lipid and was thus excluded. This resulted in one line having only two replicates, all other 183 lines have three replicates. Batch effects from lipid extraction and ESI-MS/MS profiling were analyzed and removed using PVCA [61], [62]. The count of *Wolbachia* sequence reads were normalized to the line’s genome read depth.

To compare population variances or sex variance an ANOVA accounting for between and within subject variance was used. DAPC was carried out using the adegenet package from R [63]. Population effects were removed with REML where indicated. To determine ethanol resistance LD_50_ values were estimated and analyzed using a generalized linear model with probit regression. The goodness of fit was determined with a likelihood ratio test. Differences in histogram spread were analyzed using the Wilcoxon rank sum test with continuity correction in R.

## Supporting Information

Figure S1 **Quality Control.** A: Fraction of lipid species that were excluded in quality control because they showed either no concentration across all lines or were not normally distributed. X-axis indicates the respective total that each bar was calculated in relation to. B: Same as A, but grouped by FA length with the categories odd and even. C: Frequency spectrum of estimated individual FA length. Lines are moving averages to help visualize the trends in the spectra.
(EPS)

Figure S2 **Heritabilities.** Heritability of lipid species grouped by double-bond count (A) as well as odd and even chain FAs (B) per lipid class. Lipid classes are color-coded.
(EPS)

Figure S3 **Sex differences in lipid concentration.** A: Concentration of lipid species that show sex differences (>1) in all lipid classes in males *vs*. females. Samples are color-coded by population. B: Subset of A with lipid species-specific behavior. Samples are color-coded by lipid species.
(EPS)

Figure S4 **DAPC.** A: Loading of original variables for DAPC from Figure 6 B–D. For population DAPCs, both discriminant functions are shown (Axis 1 and 2). Lipids are colored by lipid class and individual lipid species with a component loading greater than 0.2 (black line) have been annotated. (cont. = containing) B and C: Scatter plots of cross-sex DAPC eigenvector transformation as summarized in Figure 6G,H.
(EPS)

Table S1 **Tested lipid species. Containing masses, formulas and potential other lipids with similar properties.**
(XLSX)

Table S2 **Raw Lipid Concentrations.**
(CSV)

Table S3 **QTOF and FTICR MS measurements of odd-chained lipid species**
(XLSX)
